# Structure of the periplasmic adaptor protein from a major facilitator superfamily (MFS) multidrug efflux pump

**DOI:** 10.1016/j.febslet.2014.06.055

**Published:** 2014-08-25

**Authors:** Philip Hinchliffe, Nicholas P. Greene, Neil G. Paterson, Allister Crow, Colin Hughes, Vassilis Koronakis

**Affiliations:** a Department of Pathology, University of Cambridge, Tennis Court Road, Cambridge CB2 1QP, UK; b Diamond Light Source, Harwell Science and Innovation Campus, Didcot OX11 0DE, UK

**Keywords:** IM, inner membrane, RND, resistance nodulation division, MFS, major facilitator superfamily, OM, outer membrane, RMSDs, root mean square deviations, ABC, ATP-binding cassette, MP, membrane proximal, TM, transmembrane, Antibiotic resistance, Major facilitator superfamily, Multidrug efflux, Adaptor protein, Crystal structure

## Abstract

Periplasmic adaptor proteins are key components of bacterial tripartite efflux pumps. The 2.85 Å resolution structure of an MFS (major facilitator superfamily) pump adaptor, *Aquifex aeolicus* EmrA, shows linearly arranged α-helical coiled-coil, lipoyl, and β-barrel domains, but lacks the fourth membrane-proximal domain shown in other pumps to interact with the inner membrane transporter. The adaptor α-hairpin, which binds outer membrane TolC, is exceptionally long at 127 Å, and the β-barrel contains a conserved disordered loop. The structure extends the view of adaptors as flexible, modular components that mediate diverse pump assembly, and suggests that in MFS tripartite pumps a hexamer of adaptors could provide a periplasmic seal.

## Highlights

• Periplasmic adaptors are key to MFS-dependent tripartite pump efflux.
• We present the structure of *Aquifex aeolicus* EmrA, an MFS pump adaptor.
• The adaptor has an extended 127 Å long α-helical coiled-coil and disordered β-barrel loop.
• The inner membrane transporter interacting membrane proximal domain is absent in EmrA.
• This extends the modular view of adaptors, mediating diverse pump assembly.

## 1 Introduction

Tripartite efflux pumps expel a wide range of noxious molecules, including antibiotics, metals, detergents and bile salts from Gram-negative bacteria such as *Escherichia coli* and *Pseudomonas aeruginosa*, and they are major drivers of the increasing threat of multiple antibiotic resistance [1], [2]. Efflux substrates bind to an inner membrane (IM) transporter, e.g. in *E*. *coli* an ATPase like MacB or a proton antiporter like the RND (resistance nodulation division) AcrB or MFS (major facilitator superfamily) EmrB, and are delivered to the outer membrane (OM)-anchored TolC exit duct, the entrance to which projects into the periplasm [1], [2], [3]. In all pumps, an essential third component is the periplasmic adaptor (e.g. *E*. *coli* AcrA or EmrA), shown by extensive in vivo cross-linking and multidomain docking of the *E*. *coli* AcrA-AcrB-TolC RND-dependent pump to establish and stabilise interactions with both TolC and AcrB [4], [5], [6]. Structural analyses have indicated that variation among pumps is underpinned by a flexible, linearly arranged, multidomain adaptor architecture [5], [7], [8], [9], [10], [11], in which three β-sheet domains – lipoyl, β-barrel and membrane proximal – interact with the 70 Å periplasmic extension of RND IM transporters, while a fourth domain, an α-helical hairpin, establishes extensive coiled-coil interactions with the periplasmic α-helical barrel of TolC [1], [4], [5]. The recent finding that the α-hairpin is completely absent from an adaptor of the spirochete *Borrelia burgdorferi* emphasizes that adaptor variation can be substantial in effecting assembly of different pumps [10].

In the structurally uncharacterised MFS-dependent efflux pumps, such as *E*. *coli* EmrAB-TolC [12], [13], [14], [15], [16], *E*. *coli* EmrKY-TolC [17], *Vibrio cholerae* VceAB-VceC [18], [19], *Neisseria gonorrhoeae* FarAB-MtrE [20] and *Stenotrophomonas maltophilia* EmrAB-EmrC [21], component interactions must indeed differ from RND-dependent pumps as their primary sequences, together with structural characterisation of related autonomous MFS transporters such as QacA, Sge1, PepT_So_ and PepT_st_ [12], [22], [23], [24], [25], indicate that transporters in MFS-dependent tripartite machineries lack the large periplasmic extension that is important in RND pump assembly. In many organisms such as *Aquifex aeolicus*, *V*. *cholerae*, *S*. *maltophilia*, *P*. *aeruginosa*, *Cupriavidus metallidurans* and *Burkholderia pseudomallei* the tripartite MFS pump is organized in an operon with genomic structure 5′-OM pump-adaptor-MFS transporter-3′, while in *E*. *coli* only the adaptor and MFS transporter are present in an operon (*E*. *coli* TolC is separate as it is utilized by myriad MFS, ABC and RND transporters). Component interactions of MFS-dependent pumps have been evidenced in vitro, EmrA-EmrB interaction by electron microscopy [26] and EmrA-TolC using surface plasmon resonance [27]. The stoichiometry is unknown, though the adaptor EmrA, which is anchored to the IM by a single transmembrane (TM) helix [15], can form dimers and trimers in vitro [15], and electron microscopy of a reconstituted EmrAB complex suggested the formation of a ‘dimer-of-dimers’ [26]. The physiological relevance of such oligomers, e.g. how they would interact with trimeric TolC and enable substrates to bypass the periplasm through EmrAB-TolC, remains to be seen.

The structure of the closely conserved TolC exit duct has been known for 14 years [3], but an understanding of EmrAB-TolC assembly and operation requires structural information for the IM and periplasmic pump components. Here we present the structure of MFS adaptor EmrA from *A*. *aeolicus*, revealing features that appear specific to the MFS efflux pumps.

## 2 Materials and methods

### 2.1 Expression of soluble *Aquifex aeolicus* EmrA protein

To produce native, soluble *Aquifex aeolicus* EmrA (*aa*EmrA), *E*. *coli* SoluBL21 cells bearing pET24-*aa*EmrAΔTM (see Supplemental data for cloning methods) were grown at 30 °C in 2xTY medium containing 50 μg ml^−^^1^ kanamycin to *A*_600_ 0.6, when 0.5 mM IPTG was added for 16 h at 18 °C. Cells harvested by centrifugation were resuspended in 50 mM Tris pH 7.4, 400 mM NaCl, 5% glycerol, 10% MgCl_2_ supplemented with EDTA-free protease inhibitor (Roche). Cells were broken by two 30 000 psi passages through a cell disruptor. After centrifugation at 150 000×*g* for 1 h at 4 °C, supernatant was incubated with Profinity IMAC resin (Biorad) and 4 mM imidazole for 1 h at 4 °C. Resin was washed in buffer A (25 mM HEPES pH 7.5, 400 mM NaCl, 4 mM imidazole) containing 0.1% Triton X-100, then in buffer A alone. Protein was eluted in 25 mM HEPES pH 7.5, 200 mM NaCl and 400 mM imidazole. Imidazole concentration was reduced to 10 mM using an Amicon 10kDA molecular weight cut-off concentrator (Millipore). Final protein concentration was 7 mg ml^−^^1^. Selenomethionine-incorporated EmrA was produced using a metabolic inhibition protocol [28]. *E*. *coli* SoluBL21 (DE3) (Genlantis) cells bearing pET24-EmrAΔTM plasmid were grown at 37 °C to A_600_ 0.5 in M9 minimal media supplemented with 50 μg ml^−^^1^ kanamycin, 0.2% glucose, 2 mM MgSO_4_, 0.1 mM CaCl_2_ and 0.001% thiamine. At this point 100 mg L^−^^1^ threonine, lysine and phenylalanine, 50 mg L^−^^1^ leucine, isoleucine and valine and 60 mg L^−^^1^ selenomethionine were added and cells grown for a further 45 min. 0.5 mM IPTG was then added for 16 h at 18 °C. Protein was purified as native with the addition of 1 mM TCEP to all buffers. Incorporation of 2 selenomethionine residues was confirmed by mass-spectrometry. The *ec*EmrA protein lacking the TM domain was produced from plasmid pET24-*ec*EmrAΔTM and purified as native *aa*EmrA.

### 2.2 Crystallisation, structure determination and analysis

Crystallisation of *aa*EmrA was conducted using sitting drop vapour diffusion at 15 °C in CrysChem 24 well plates (Hampton Research). Drops were formed by mixing 2 μl of protein solution with 2 μl of crystallisation reagent (100 mM MES pH 6.5, 100 mM MgCl_2_, 10% isopropanol, 8% PEG4000) and equilibrated against 500 μl. Crystals grew to maximum size (0.8 mm × 0.2 mm × 0.2 mm) in seven days, and were cryoprotected by stepwise addition of cryoprotectant (100 mM MES pH 6.5, 50 mM MgCl_2_, 5% isopropanol, 10% PEG4000, 42.5% MPD), before being looped and flash-frozen in liquid nitrogen. Diffraction data were collected at 100 K on beamline I24 (Diamond Light Source, UK). X-ray data sets were indexed and integrated using XDS [29] and scaled using Aimless in the CCP4 suite [30]. Crystallographic phases for the 3.5 Å selenomethionine dataset were obtained by the Single-wavelength Anomalous Dispersion (SAD) method, with two selenium sites identified using ShelxD [31]. Sites were refined and an initial 3.5 Å resolution density modified map created using Autosharp. Density modification was then performed in Resolve using the isomorphous 2.85 Å high-resolution data, maintaining the same ‘free’ reflection list and using the initial SeMet sites and electron density map from the SAD data. An initial model of EmrA was built with AutoBuild in Phenix. The structure was completed with iterative rounds of manual model-building with Coot [32] and refinement in Phenix [33]. Structure validation was assisted by Molprobity [34] and Procheck [35]. The final model encompasses one molecule of EmrA (residues 27–321 and 343–373), 6 water molecules, 2 isopropanol molecules and a magnesium ion, with no Ramachandran outliers. The atomic coordinates and structure factors have been deposited in the Protein Data Bank (http://www.rcsb.org/), PDB ID: 4TKO. Root mean square deviations (RMSDs) were calculated over C_α_ atoms aligned using superpose in the CCP4 suite [36]. The colour scheme of conserved residues in Supplemental Fig. 4 was generated with CONSURF [37], [38]. Figures were prepared using PyMol [39].

## 3 Results and discussion

### 3.1 Crystallisation and structure determination of *Aquifex aeolicus* EmrA

Sequence analysis of EmrA proteins (360–423 amino acids in length, Supplemental Fig. 1) reveals the N-terminus consists of a short (6–32 amino acid), unconserved cytoplasmic domain preceding a TM helix, analogous to the AcrA lipid attachment, that anchors the adaptor in the IM. To facilitate structural studies, we cloned and expressed EmrA from *E*. *coli* and 5 homologues, engineered to lack the TM helix (see Supplemental Methods). Most either failed to crystallise or otherwise produced non-diffracting crystals, but we were able to solve the structure of *Aquifex aeolicus* EmrA (*aa*EmrA) from a construct encompassing residues 26 onwards of the 374 residue mature sequence. Native *aa*EmrA crystallised in space group I4_1_22 with a 78% solvent content and one molecule in the asymmetric unit. Phasing using molecular replacement with adaptor homologues was unsuccessful. Selenomethionine derivatised *aa*EmrA crystallised as native and phases were calculated using a 3.5 Å resolution single-wavelength anomalous diffraction (SAD) dataset (Table 1) and the positions of two methionine residues and an initial density modified map were determined. These were used as starting points to calculate the electron density map of an isomorphous 2.85 Å resolution dataset into which an atomic model could be built (Table 1). The final refined model contains residues 27–321 and 343–373 with an *R*_work_ and *R*_free_ of 20.8 and 24.8, respectively. Residues 26, 322–342 and 374 could not be built due to poorly defined electron density.

**Table 1:** Data collection and refinement statistics.

	*aa*EmrA – *SeMet*	*aa*EmrA – native
*Data collection*		
Space group	*I*4_1_22	*I*4_1_22

Cell dimensions		
* a*, *b*, *c* (Å)	81.15, 81.15, 540.24	81.37, 81.37, 541.70
α, β, γ (°)	90, 90, 90	90, 90, 90

Wavelength	0.96861	1.03836
Resolution (Å)	39.34–3.46 (3.79–3.46)	29.67–2.85 (3.00–2.85)
*R*_merge_	0.170 (0.640)	0.171 (0.476)
*I*/σ*I*	14.8 (4.8)	9.70 (3.30)
Completeness (%)	99.5 (98.0)	99.98 (100.00)
Redundancy	16.6 (16.8)	11.9 (11.3)

*Refinement*		
Resolution (Å)		29.67–2.85
No. reflections		22 150
*R*_work_/*R*_free_		20.8/24.8
No. atoms		
Protein		2654
Ligand/ion		9
Water		6

*B*-factors (Å^2^)		
Protein		71.05
Ligand/ion		64.00
Water		52.2

R.m.s. deviations		
Bond lengths (Å)		0.008
Bond angles (°)		0.99

Ramachandran plot		
Outliers (%)		0
Allowed (%)		1.86
Favoured (%)		98.14

Values in parentheses are for highest-resolution shell.

### 3.2 EmrA lacks a membrane proximal domain present in adaptors from distinct pumps

**Fig. 1:**
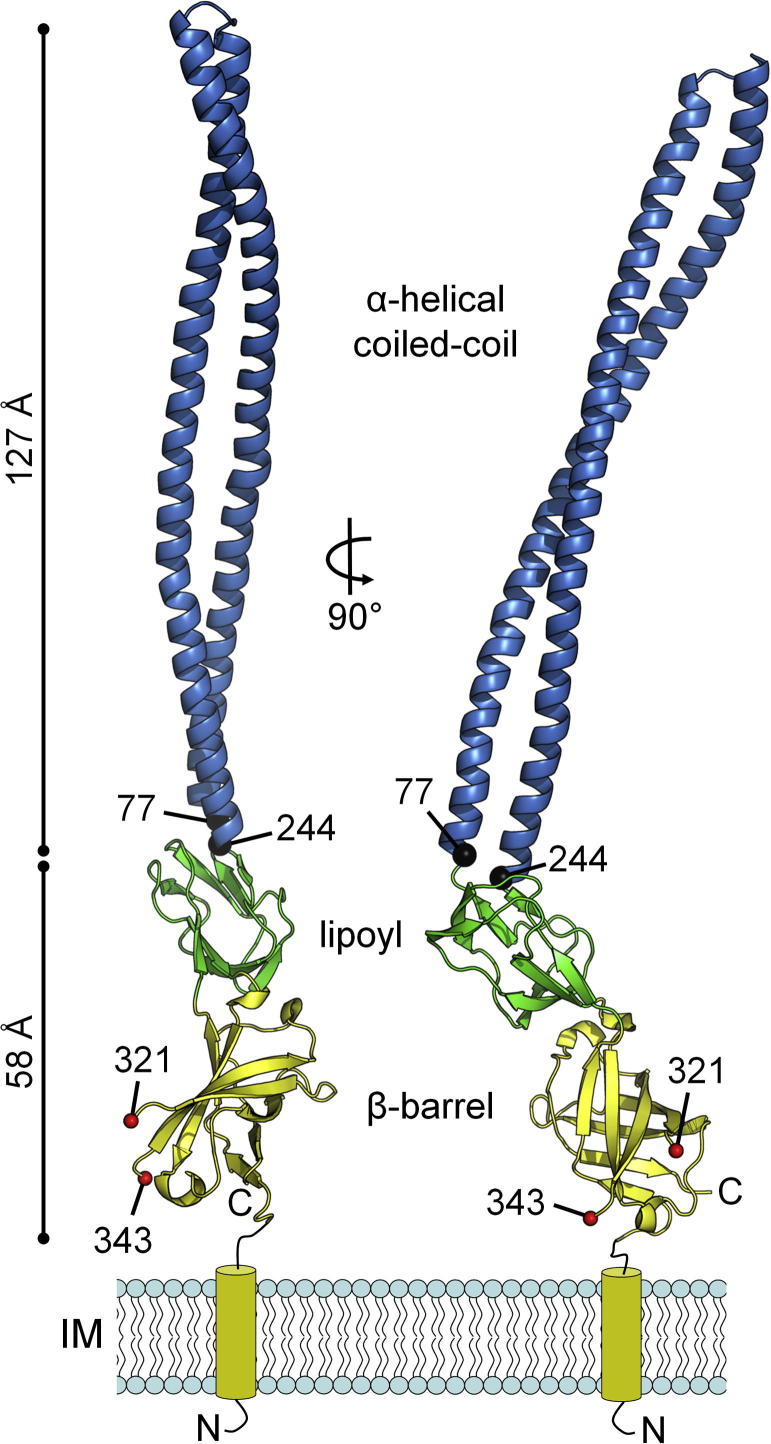
Overall structure of *Aquifex aeolicus* EmrA. EmrA is coloured by domain in yellow (β-barrel), green (lipoyl) and blue (α-helical coiled coil). The β-barrel contains the N and C-termini (labelled) and anchors EmrA in the inner membrane (IM) via an N-terminal TM helix (yellow tube). Due to poorly defined density for residues 322–342, there is a break in the β-barrel with residues 321 and 343 (red spheres) indicating the beginning and end of modelled residues for this loop.

The structure contains three linearly arranged domains, an α-helical hairpin, a lipoyl domain and a β-barrel domain (coloured blue, green and yellow in Fig. 1). While this domain organisation is similar to other structurally characterised adaptors, most obviously *aa*EmrA lacks the membrane proximal (MP) domain identified to date in all 6 RND and ATP-binding cassette (ABC) adaptor crystal structures [7], [8], [9], [10], [11], [40]. Therefore, distinctively for periplasmic adaptors, the β-barrel is proximal to the IM, anchored via its N-terminal TM helix. Sequence alignment (Supplemental Fig. 1) of EmrA homologues from 13 diverse bacteria indicates the lack of an MP domain is not unique to *A*. *aeolicus*. Some EmrA adaptors have non-conserved C-terminal extensions (19 and 41 residues in *V*. *cholerae* VceA and *ec*EmrA, respectively) but these extra residues are insufficient to form an MP domain, which are usually 84–108 residues long. In fact, the C-terminal residues of *ec*EmrA are predicted to form a short α-helix [15], not the usual β-roll topology of MP domains. In addition, the N-terminal TM helix is directly attached to the β-barrel domain in all EmrA homologues, positioning this domain closest to the membrane and indicating the extra C-terminal residues do not form a fourth EmrA domain adjacent to the membrane.

In contrast to EmrA, the membrane attachment site in RND and ABC adaptors is located at the MP domain (e.g. an N-terminal lipoyl group in RND AcrA or an N-terminal TM helix in ABC MacA). In RND-dependent tripartite pumps, the MP domain is known to make extensive interactions with the periplasmic extension of IM transporters [5], [41]. This MP domain would not be required for EmrA-EmrB interactions in MFS pumps as there is no such periplasmic extension of the IM EmrB transporter.

### 3.3 An exceptionally long α-helical coiled-coil in the MFS adaptor EmrA

The *aa*EmrA anti-parallel, two-stranded, α-helical coiled-coil domain is 168 residues and 127 Å long with 11 heptad repeats per helix. The N-terminal helix consists of residues 77–160, and the C-terminal helix residues 165–244 (Fig. 1). The coil is non-ideal due to positively charged residues in its hydrophobic core, i.e. K84, K98, R130, R182 and K119 reside in normally hydrophobic A and D heptad positions. In addition, the heptad repeat pattern is disrupted by a four residue insertion in the N-terminal helix (residues 112–115) and a three residue insertion in the C-terminal helix (residues 210–212), approximately 70 Å from the tip of the structure, and close to the centre of the coiled-coil. This disrupts the knobs-into-holes packing, resulting in a weakened inter-helical interaction in this region of the coil. Heptad shifts, also called “stutters” and “stammers”, are usually found in α-fibrous proteins with an extended coiled-coil structure such as myosin and fibrinogen, where it is suggested they may serve as points of flexibility for extremely long coiled-coils [42], [43]. The shifts here likely result in relative flexibility in this region, evident as high C_α_ *B*-factors over the centre of the *aa*EmrA coiled-coil (an average of 124 Å^2^ over residues 102–125 and 200–222 compared to 90 Å^2^ over the entire α-hairpin and 71 Å^2^ over the whole structure, Supplemental Fig. 2A). The coil also contains a large number of lysine residues (21% over coil residues 77–244 compared to 14% over the full structure) resulting in a positive charge distribution (the electrostatic charge over the *aa*EmrA surface is presented in Supplemental Fig. 2B), particularly evident in the centre of the coil (residues 119–136 and 184–202 contain 31% lysine residues) close to the heptad phase shifts.

**Fig. 2:**
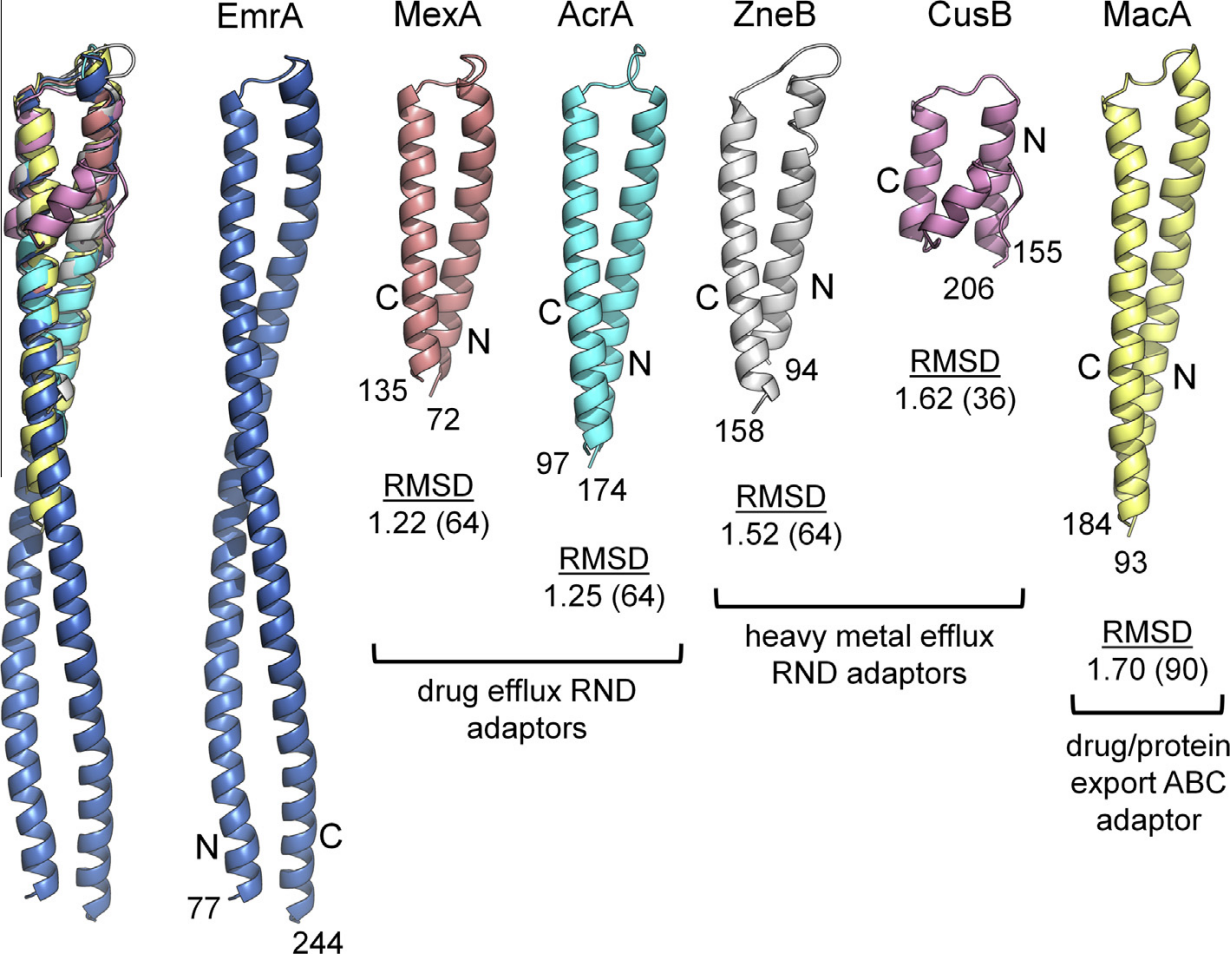
Structural alignment of adaptor α-helical coiled-coils. *Left*, overlay of *Aquifex aeolicus* EmrA (blue) with adaptors *P*. *aeruginosa* MexA (red, PDB: 2V4D), *E*. *coli* AcrA (cyan, 2F1M), *C*. *metallidurans* ZneB (grey, 3LNN), *E*. *coli* CusB (purple, 3OOC) and *E*. *coli* MacA (yellow, 3FPP). *Right*, adaptors aligned with the RMSDs (in Å) of each adaptor α-hairpin to EmrA, calculated using superpose in the CCP4 suite, indicated below the respective structure (number of aligned residues in brackets). N- and C- terminal residues are labelled.

An alignment of structurally characterised adaptors to date (Supplemental Fig. 3) highlights the differences in the length of adaptor α-hairpins. The 127 Å long coiled-coil of *aa*EmrA is at least twice as long as the α-hairpins of the multidrug RND adaptors AcrA and MexA (58 Å and 47 Å long, respectively [11], [40]), the heavy metal efflux RND adaptor ZneB (52 Å long [7]) and the drug efflux ABC adaptor MacA (67 Å long [9]) (Fig. 2). The length of the *aa*EmrA α-hairpin is even more pronounced when compared to the 27 Å long α-hairpin of the heavy metal efflux adaptor CusB, which contains an unusual three-helix bundle [8], and the recently published structure of BesA, an RND adaptor from the Spirochaete *B*. *burgdorferi*, which has no α-hairpin at all [10]. CusB therefore remains the only characterised adaptor that forms a more elaborate three-stranded coiled-coil instead of the typical two-stranded coils found in all other adaptors, including EmrA. Primary sequence analysis of EmrA homologues (Supplemental Fig. 1) suggests the minimum EmrA α-hairpin length is 118 residues (e.g. *ec*EmrA), while coils similar in length to *aa*EmrA are predicted in pathogenic *V*. *cholerae* VceA (∼146 residues/∼115Å long) as well as *Thermodesulfobium narugense* (∼130 residues/∼95Å long).

Structural alignment of the *aa*EmrA adaptor α-hairpin with those from MexA, ZneB, AcrA, CusB and MacA results in low RMSDs (1.22–1.70 Å), with the hairpins superposed to the top half of the *aa*EmrA α-hairpin (residues 116–206) (Fig. 2). The α-hairpins of MexA and AcrA are known to bind to the cognate OM exit duct TolC [1], suggesting there is close structural topology in the TolC interacting region of adaptors, including EmrA. It is therefore likely the pseudo three-fold symmetry created by the coiled-coil interactions of TolC with RND adaptors [4], [5] are also key to EmrA-TolC interactions, and suggests EmrA may function similarly to RND adaptors in stabilising TolC opening [1], [5], [44].

### 3.4 Closely conserved β-barrel and lipoyl domains

**Fig. 3:**
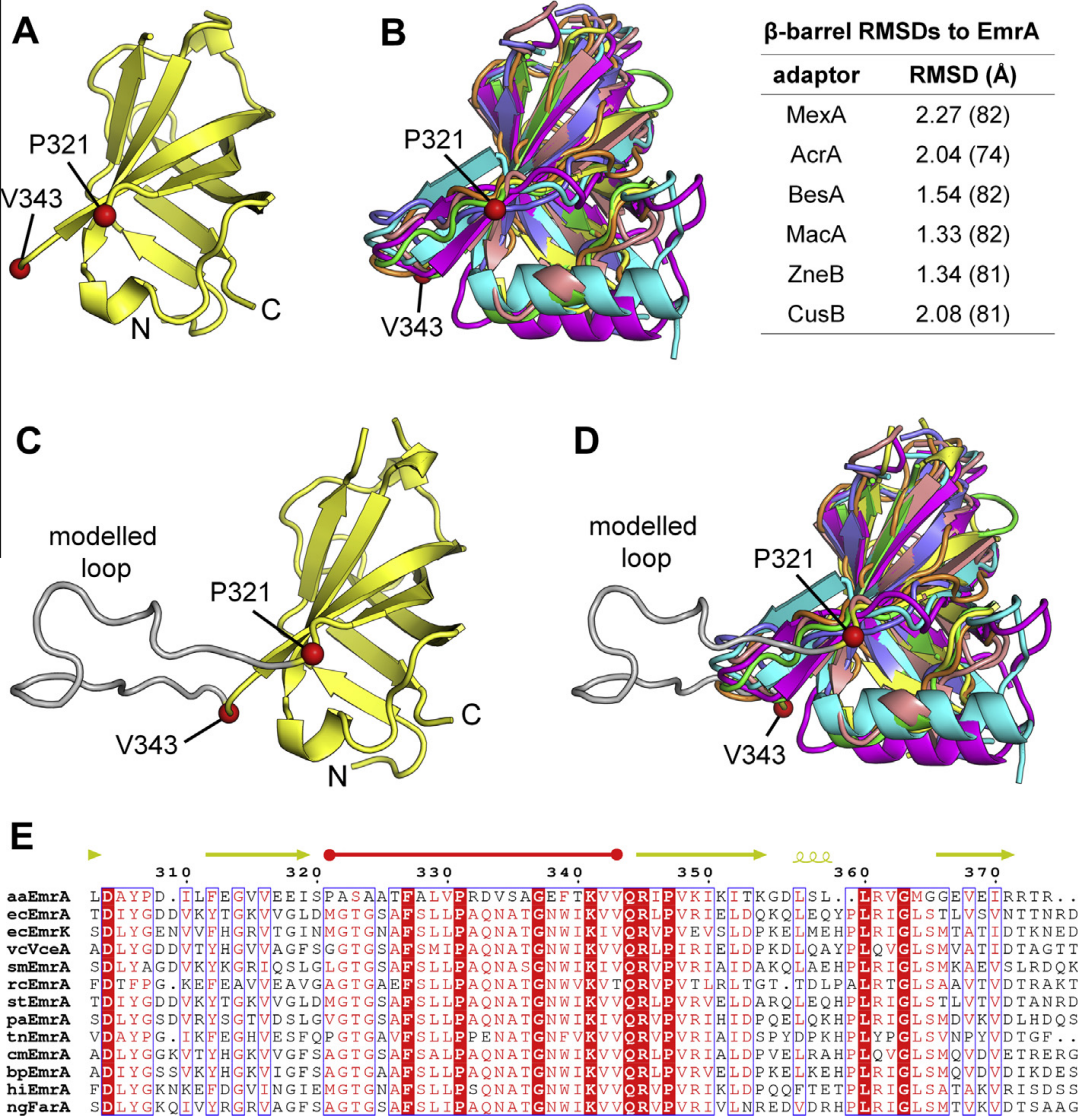
Structural alignment of adaptor β-barrels. (A) EmrA β-barrel. Red spheres indicate modelled residues (labelled) at the beginning and end of the disordered region. (B) Superposition of adaptor β-barrels. EmrA (yellow), MexA (cyan), AcrA (pink), ZneB (blue), CusB (red), BesA (green) and MacA (orange) were aligned using superpose in the CCP4 suite. RMSDs between β-barrels are tabulated to the right (number of aligned residues in brackets). (C) Disordered loop in EmrA. The disordered residues have been modelled (grey loop) as a simple loop projecting out from the β-barrel. (D) Superposition of adaptor β-barrels, highlighting the disordered loop region. Coloured as in (B) and (C). (E) Sequence alignment of EmrA homologues. Alignment was performed over the whole sequence, but for clarity only the region covering the disordered loop is shown. Sequences are from *Aquifex aeolicus* (aaEmrA), *Escherichia coli* (ecEmrA and ecEmrK), *Vibrio cholerae* (vcVceA), *Stenotrophomonas maltophilia* (smEmrA), *Rhodobacter capsulatus* (rcEmrA), *Salmonella* Typhimurium (stEmrA), *Pseudomonas aeruginosa* (paEmrA), *Thermodesulfobium narugense* (tnEmrA), *Cupriavidus metallidurans* (cmEmrA), *Burkholderia pseudomallei* (bpEmrA), *Haemophilus influenza* (hiEmrA), *Neisseria gonorrhoeae* (ngFarA). Strictly conserved residues are boxed in white on a red background and highly conserved residues are boxed in red on a white background. Secondary structure is indicated above and coloured by domain as Fig. 1.

The lipoyl and β-barrel domains are the only two domains that are conserved in structurally characterised adaptors from different pumps and organisms (EmrA lacks an MP domain and *Borrelia* BesA lacks an α-hairpin, see Supplemental Fig. 3). The lipoyl domain is structurally similar in all adaptors including EmrA (RMSDs 1.24 Å, 1.19 Å, 1.70 Å, 1.04 Å, 1.22 Å, 1.29 Å for AcrA, BesA, CusB, MacA, ZneB and MexA, respectively). Likewise, the EmrA β-barrel domain (Fig. 3A) shares structural topology with the β-barrel domains of adaptors BesA, MexA, AcrA, CusB, ZneB and MacA (RMSDs 1.54 Å, 2.27 Å, 2.04 Å, 2.08 Å, 1.34 Å and 1.33 Å, respectively) (Fig. 3B). However, the *aa*EmrA β-barrel contains 21 disordered residues, unobserved due to a break in the electron density (residues 322–342). Modelling of this region indicates it can form a 23 Å long loop, approximately the same length as the β-barrel strands (Fig. 3C). In other adaptors the equivalent loop is less than half the size, typically 7 residues long, highlighted in the β-barrel superposition in Fig. 3D. Alignment of EmrA proteins from diverse bacteria reveals a number of highly conserved residues in this long loop (Fig. 3E), including strictly conserved residues F327, P331, G337 and K341 (*aa*EmrA numbering). The loop is one of the most conserved regions in the EmrA structure and is not seen in any of the other EmrA β-barrel loops (Supplemental Fig. 4) or in other adaptors, indicating it may have a functional role in MFS pumps.

### 3.5 No EmrA ligand binding detected by isothermal titration calorimetry or co-crystallisation

The initial observation that adaptor β-barrel topology is also found in domains involved in ligand binding prompted the idea adaptors may have a role in substrate engagement [40]. Indeed, this seems to be the case for the heavy metal efflux pump adaptors ZneB and CusB in which crystal structures have revealed Zn and Cu ions bound in the flexible linker region between the β-barrel and membrane proximal domain [7], [8]. Substrate binding to *ec*EmrA lacking the TM helix was previously inferred from changes in the degree of iodide-induced quenching of intrinsic protein fluorescence upon addition of hydrophobic substrates nalidixic acid, CCCP or DNP [15]. Here, we used isothermal titration calorimetry (ITC) to investigate drug binding to constructs of *ec*EmrA or *aa*EmrA lacking the TM domain. The titration of 1 mM DNP and 10 mM nalidixic acid to 0.1 mM *ec*EmrA or *aa*EmrA did not result in a detectable interaction between substrate and adaptor (Supplemental Fig. 5). Neither could we observe any difference density consistent with bound substrates in co-crystal trials of 0.5 mM CCCP and 0.5 mM DNP with *aa*EmrA (data not shown). If EmrA does facilitate drug transfer through the tripartite pump by binding substrates as suggested [15], it is not detectable by ITC or crystallography.

### 3.6 Implications of the EmrA adaptor structure for assembly of the MFS-dependent pump

**Fig. 4:**
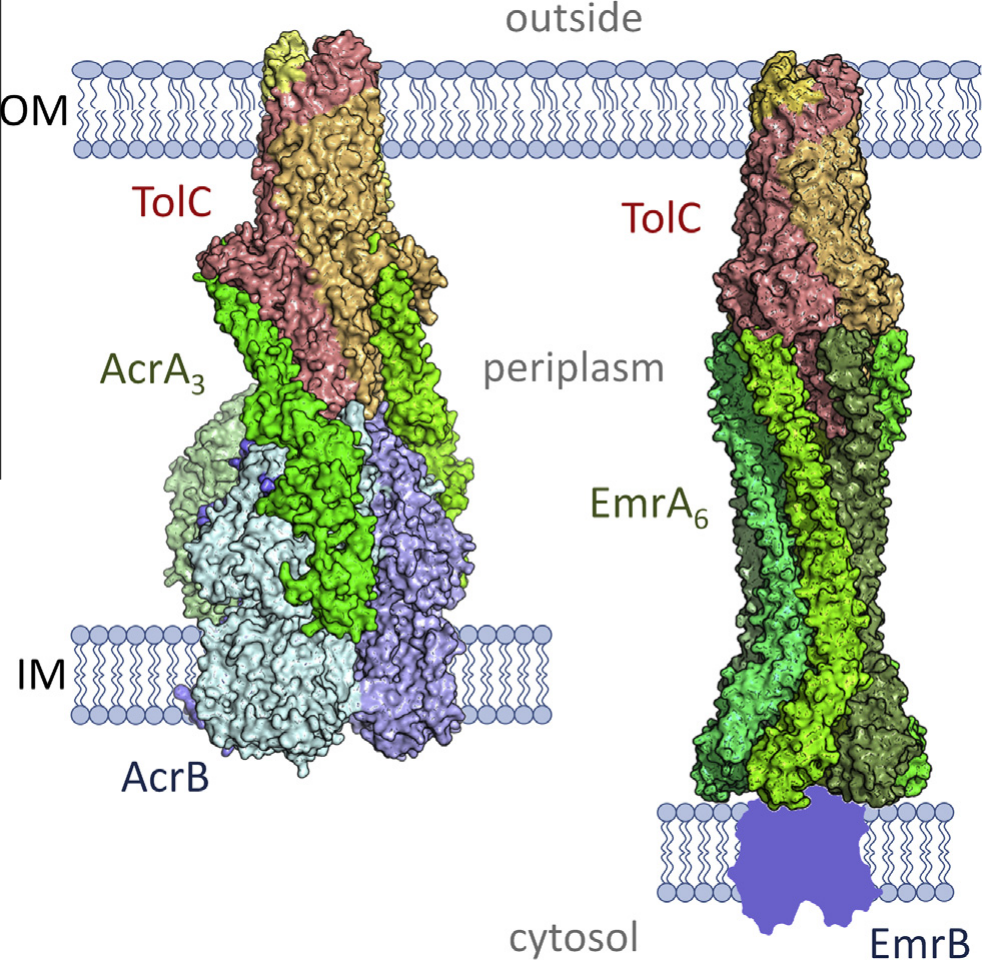
Putative assembly of the EmrAB-TolC pump. *Left*, assembled *E*. *coli* TolC (red)-AcrA (green)-AcrB (blue) pump, based on in vivo site-specific cross-linking and data-based multidomain docking. *Right*, putative assembly of Aquifex EmrA (green)-EmrB (blue, corresponding to an outline of the homologous 14 TM MFS transporter PepT_So_[25])-TolC (red). To form a seal against the periplasm for drugs to bypass inner and outer membranes (IM and OM, respectively) we have modelled a ring of six adaptors.

Tripartite efflux pumps span both inner and outer membranes, providing a continuous seal for drugs to bypass the periplasm. Extensive in vivo cross-linking analyses have shown that in AcrAB-TolC a 170 Å long periplasmic seal can be provided by close fit of IM and OM components (AcrB and TolC, respectively) [6], [45], [46] stabilised by interactions with the adaptor (Fig. 4, *left*) [1], [4], [5]. In EmrAB-TolC, the inner membrane MFS component does not contain significant periplasmic structure, so it follows the seal would be provided by the adaptor and TolC α-barrel. If six EmrA molecules are modelled into a ring, formed by lipoyl and β-barrel interactions, and close side-to-side packing of the lower EmrA α-hairpin regions (Fig. 4, *right*) that are equivalent to the upper TolC α-barrel, small gaps between β-barrel domains can only be sealed by modelling the disordered 21 β-barrel residues. In addition, the TolC-interacting regions of the α-hairpins naturally diverge in the adaptor hexamer, precluding a tip-to-tip TolC-EmrA interaction. Only by docking the TolC open form [44] into the EmrA hexamer’s upper half could we form a sealed periplasmic efflux channel, with a 225 Å periplasmic spanning distance (Fig. 4, *right*). While this length is similar to the cryo-electron microscopy model of the AcrAB-TolC complex formed by non-natural linked fusion proteins [47], it is longer than the in vivo data driven model of AcrAB-TolC (170 Å, Fig. 4, *left*) and the in vitro bipartite MexA-OprM cryo-tomography model (210Å) [1], [5], [48]. In our speculative EmrAB-TolC model the EmrA α-hairpin tips interact with the TolC periplasmic entrance coils, similar to previous in vivo and in vitro observations of adaptor-TolC coiled-coil interactions in RND-dependent tripartite machineries [4], [5]. EmrA-TolC interactions would be aided by inter-domain movement around the adaptor’s flexible linker regions [5], [10], [11], [49], previously identified as key to tripartite pump assembly [1], [5]. This hexameric ring of adaptors is also observed in the co-crystal structure of an adaptor-transporter complex (CusB-CusA [41]) and the in vitro cryo-electron microscopy based model of an AcrAB-TolC complex formed by linked fusion proteins [47], suggesting the seal in these systems could also be provided by a ring of 6 adaptors, stabilised by interactions through the β-barrel and lipoyl domains. As for adaptor-transporter interactions, the single TM helices of EmrA would seem likely to form substantial contacts with the predicted 14 TM helices of EmrB. Indeed, the putative ring of β-barrels from the six EmrA adaptors would have an internal diameter similar to the size of monomeric structurally characterised 14 TM MFS adaptors (oligopeptide transporters PepT_So_ [25] and PepT_St_ [23]), suggesting the single TM helices of the 6 adaptors could interact with monomeric EmrB by forming a 20-helix bundle in the IM. While we require further experimental evidence to support the model, this arrangement would contrast with the ‘dimer-of-dimers’ suggested by electron microscopy of purified EmrA and EmrB [26].

## Appendix A Supplementary data

Supplementary data 1 This document contains supplementary data.
