# Supplementary material for: Structure of the periplasmic adaptor protein from a major facilitator superfamily (MFS) multidrug efflux pump
Source: FEBS Lett. 2014 Aug 25;588(17):3147–53. doi: 10.1016/j.febslet.2014.06.055 (PMC4158417; doi:10.1016/j.febslet.2014.06.055)
Supplement: Supplementary data 1 — This document contains supplementary data. [file mmc1.pdf]

Supplemental Methods and Figures for

Structure of the periplasmic adaptor protein from a major facilitator superfamily (MFS) multidrug efflux pump

Philip Hinchliffe, Nicholas P. Greene, Neil G. Paterson[1]*, Allister Crow,*

Colin Hughes, Vassilis Koronakis[2]

Department of Pathology, University of Cambridge, Tennis Court Road, Cambridge, CB2 1QP, UK

[1]Diamond Light Source, Harwell Science and Innovation Campus, Didcot, OX11 0DE, UK

[2]Corresponding author: Vassilis Koronakis, Tel: +44 (0)1223 339766; E-mail: vk103@cam.ac.uk

Supplemental Methods

Cloning soluble EmrA homologues

To express soluble *Aquifex aeolicus* EmrA (*aa*EmrA) we removed the transmembrane (TM) helix by amplifying *emrA* codons 27-374 from *Aquifex aeolicus* genomic DNA with forward and reverse primers (5′-GCGCCATATGAAACACAGAATTGAGTATGCCATAACAAACG-3′, 5′-GCGCTCGAGTCTCGTTCTCCTTATCTCAACCTCACCGC-3′). The product was digested with NdeI/XhoI and cloned into pET24 (Novagen). The resultant plasmid was designated pET24-*aa*EmrAΔTM. Plasmid pET24-*ec*EmrAΔTM expressing soluble *E.coli* EmrA (codons 48-390, lacking the TM domain) was produced by amplification of genomic DNA with forward (5′-GCGCCATATGCACTTCGAAGAAACCGATGACGCATACG-3′) and reverse (5′-GCGCCTCGAGGCCAGCGTTAGCTTTTACGATATCG-3′) primers, digestion and ligation as pET24-*aa*EmrAΔTM. We cloned EmrA homologues from *Rhodobacter capsulatus, Pseudomonas aeruginosa, Salmonella enterica* Typhimurium and *Thermodesulfobium narugense* with a similar strategy.

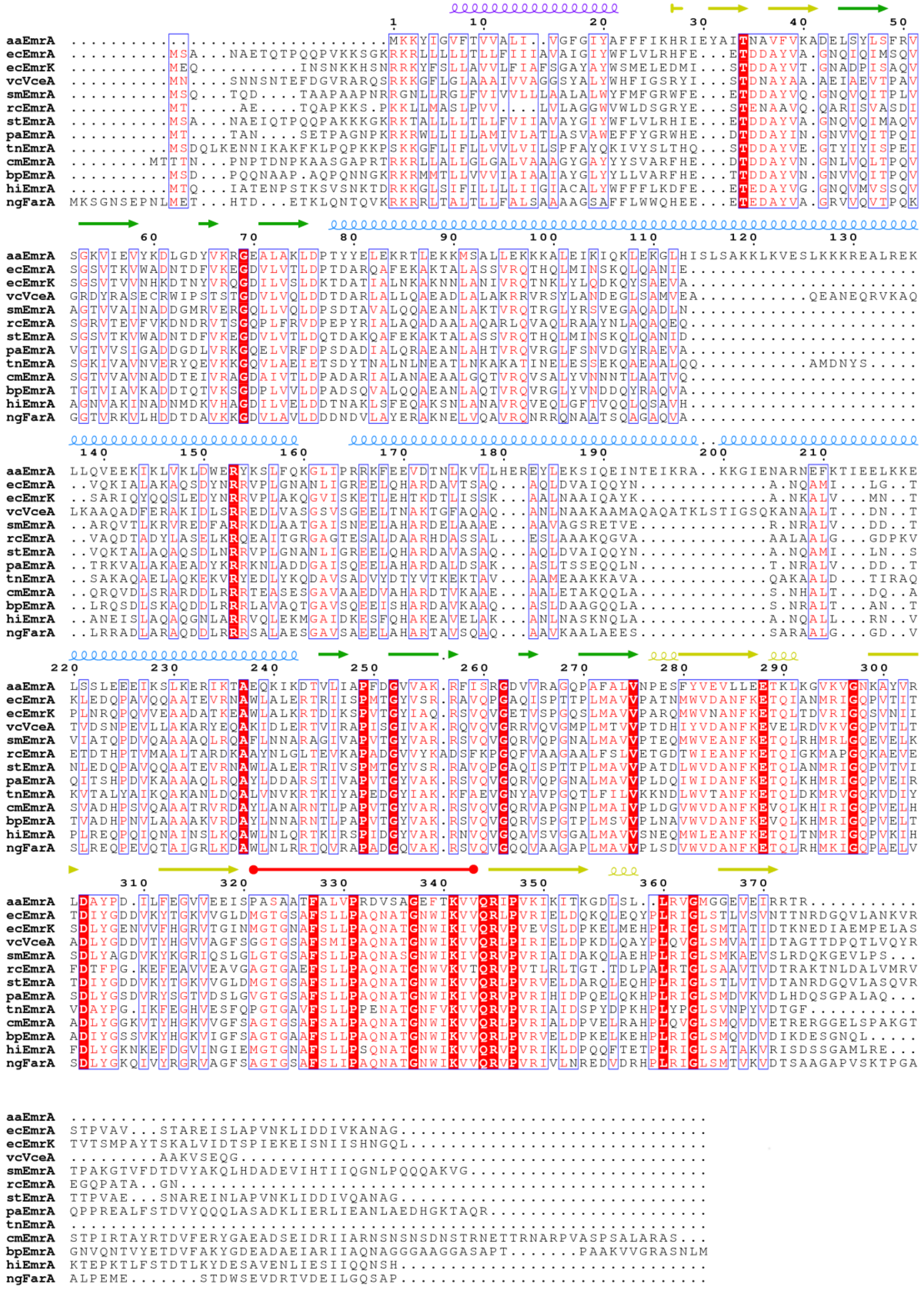

Supplemental Figure 1. Sequence alignment of EmrA homologues.

Amino acid sequence alignment of EmrA from *Aquifex aeolicus* (aaEmrA), *Escherichia coli* (ecEmrA and ecEmrK), *Vibrio cholerae* (vcVceA), *Stenotrophomonas maltophilia* (smEmrA), *Rhodobacter capsulatus* (rcEmrA), *Salmonella* Typhimurium (stEmrA), *Pseudomonas aeruginosa* (paEmrA), *Thermodesulfobium narugense* (tnEmrA), *Cupriavidus metallidurans* (cmEmrA), *Burkholderia pseudomallei* (bpEmrA), *Haemophilus influenza* (hiEmrA), *Neisseria gonorrhoeae* (ngEmrA). Strictly conserved residues are boxed in white on a red background and highly conserved residues are boxed in red on a white background. Secondary structure is indicated above and coloured according to domain (purple, predicted transmembrane helix; yellow, β-barrel; green, lipoyl; blue, α-helical coiled-coil; red, β-barrel disordered loop).

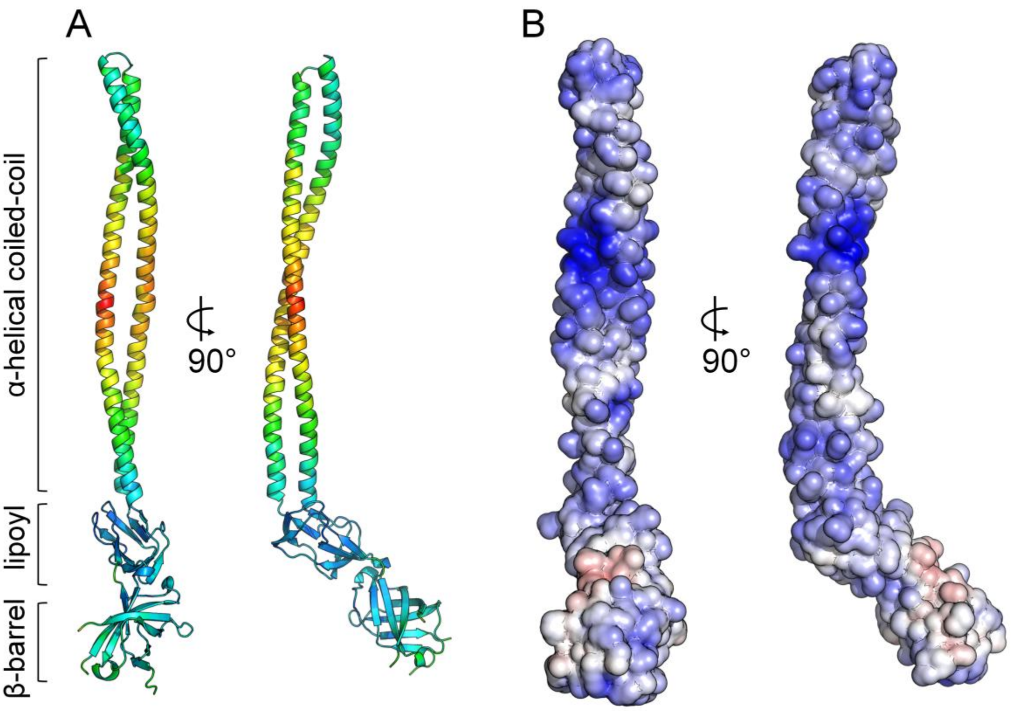

Supplemental Figure 2. Disorder and electrostatic distribution over the EmrA structure.
(A) B-factor distribution over the EmrA structure. The crystal structure of Aquifex EmrA is coloured according to C_α B-factors, from blue (low B-factor) to red (high B-factor).
(B) EmrA electrostatic surface. The EmrA surface is shown with positively and negatively charged surface areas coloured blue and red, respectively. Electrostatic surface potentials were calculated using the program APBS and contoured at ± 9 kT/*e*.

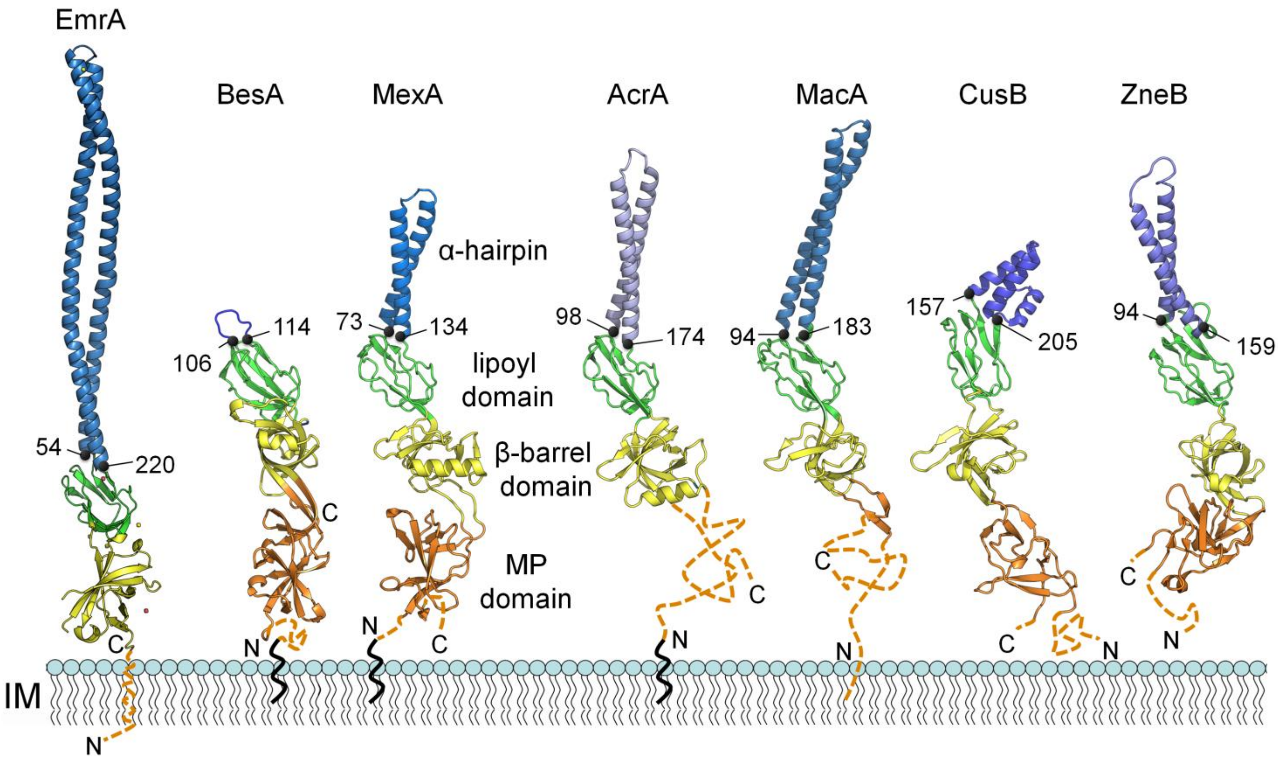

Supplemental Figure 3. Alignment of structurally characterised periplasmic adaptors.
EmrA structure compared to structurally characterised drug efflux pump adaptors BesA (*B.burgdorferi*), MexA (*P.aeruginosa*), AcrA (*E.coli*) and MacA (*E.coli*) and the metal efflux pump adaptors CusB (*E.coli*) and ZneB (*C.metallidurans*). Adaptors are coloured by domain, blue (α-helical coiled-coil), green (lipoyl), yellow (β-barrel) and orange (membrane proximal, MP). Dotted orange lines indicate unobserved adaptor C- and N- terminal regions.

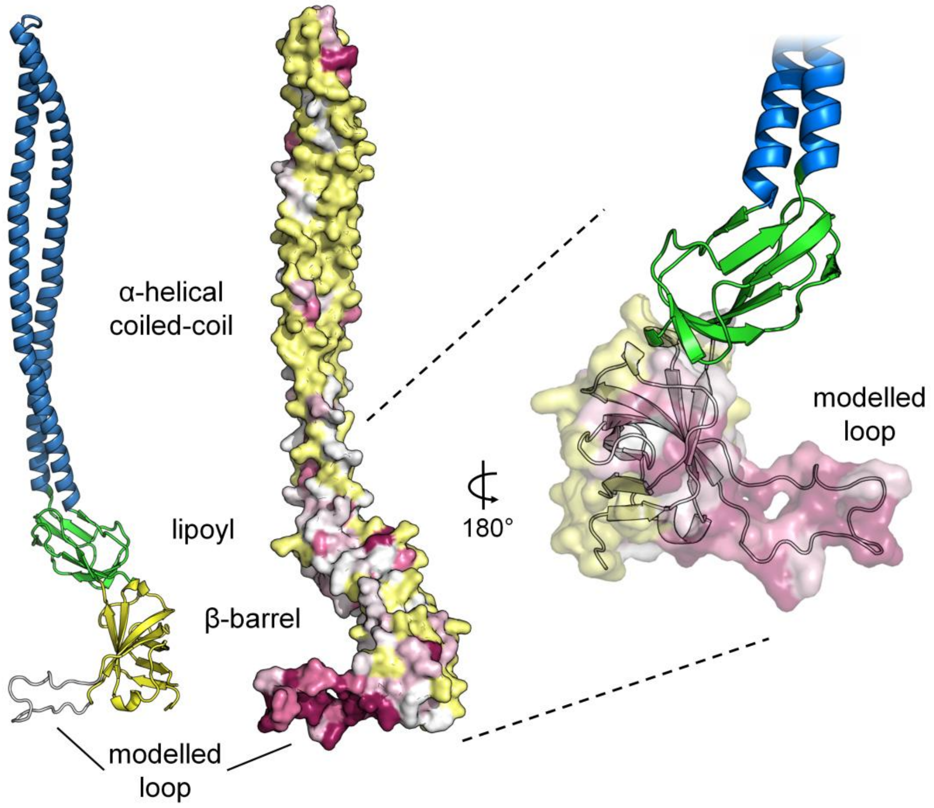

Supplemental Figure 4. Sequence conservation over EmrA.
Left, structure of EmrA coloured by domain as in main text Figure 1. The disordered residues 322-342 have been modelled as a loop (grey) projecting out from the β-barrel. ***Centre***, surface representation of EmrA with conserved amino acids coloured from highest conserved residues (pink) to lowest conserved residues (yellow). ***Right***, a close up of the β-barrel, rotated 180° from the centre view.

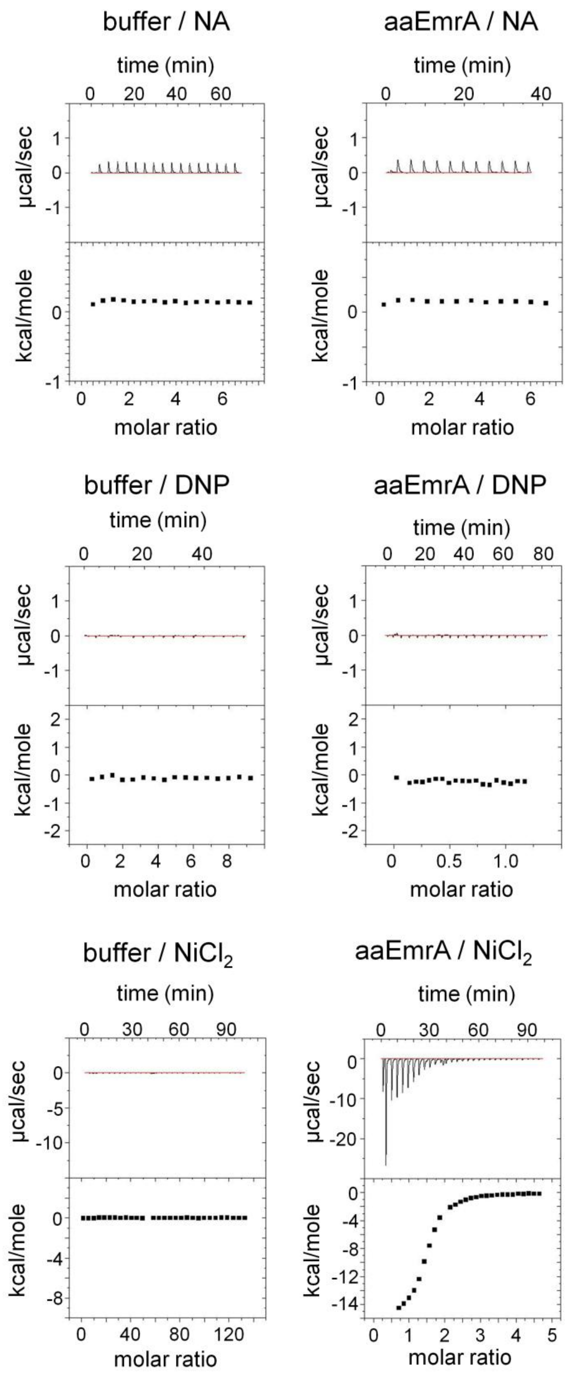

Supplemental Figure 5. Isothermal titration calorimetry properties of EmrA titrated with known EmrAB substrates.

Soluble constructs of 0.1mM *E.coli* and *A.aeolicus* EmrA (*ec*EmrA and *aa*EmrA, respectively) were exchanged into 20mM HEPES pH8.0, 150mM NaCl on a superdex 200 gel filtration column and concentrated to 0.1mM in an Amicon 10kDA molecular weight cut-off concentrator (Millipore). They were then titrated with 10mM nalidixic acid (NA) or 1mM DNP in the same buffer. ITC measurements were carried out at 25°C using a VP-ITC MicroCalorimeter (Microcal). Protein (0.1mM) was placed in the 1400µl calorimeter cell and 8µl of titrant was injected over 16s with 200s intervals between each injection. Titrant into buffer alone was used as a negative control. As a positive control, binding of 10mM $NiCl_2$ to the his-tags of *ec*EmrA and *aa*EmrA was analysed using the same parameters. Data were analysed using Origin software (Microcal). In each case, the upper panel shows raw energy changes during the titration (time), while the lower panel presents the derived integrated total energy changes as a function of the molar ratio of the interactants.
